# Mingjing granule, a traditional Chinese medicine in the treatment of neovascular age-related macular degeneration: study protocol for a randomized controlled trial

**DOI:** 10.1186/s13063-021-05025-x

**Published:** 2021-01-19

**Authors:** Yamin Li, Lina Liang, Torkel Snellingen, Kai Xu, Yun Gao, Fengmei Zhang, Chengwei Guo, Tao Zuo, Fengming Liang, Xiaoping Yao, Xueyan Yang

**Affiliations:** 1 Eye Hospital, China Academy of Chinese Medical Sciences, 33 Lugu Road, Shijingshan District, Beijing, 100040 China; 2 Aurora Eye Research Foundation, St. Olavsgate 4, 3126 Tønsberg, Norway; 3 Henan Province Hospital of Traditional Chinese Medicine/The Second Affiliated Hospital of Henan University of Chinese Medicine, Zhengzhou, 450002 China; 4 The Affiliated Hospital of Shandong University of Traditional Chinese Medicine /Shandong Province Hospital of Traditional Chinese Medicine, Jinan, 250011 China; 5 The Second Affiliated Hospital of Liaoning University of Traditional Chinese Medicine, Shenyang, 110034 China; 6 The First Teaching Hospital of Tianjin University of Traditional Chinese Medicine, Tianjin, 300110 China; 7 Shenzhen Traditional Chinese Medicine Hospital, Shenzhen, 518033 China; 8 Foshan Hospital of Traditional Chinese Medicine, Foshan, 528500 China

**Keywords:** nAMD, Mingjing granule, Randomized controlled trial

## Abstract

**Background:**

Neovascular age-related macular degeneration (nAMD) is the most common cause of irreversible vision loss and blindness among the older people aged 50 and over. Although anti-vascular endothelial growth factor (anti-VEGF) therapies have resulted in improving patient outcomes, there are limitations associated with these treatments. In China, traditional Chinese medicine (TCM) has been used to treat eye diseases for more than 2000 years. Previous studies have shown that TCM may be beneficial for nAMD patients. However, explicit evidence has not been obtained. The purpose of the present trial is to examine the efficacy and safety of the Mingjing granule, a compound Chinese herbal medicine, for nAMD patients.

**Methods/design:**

This is a double-blind, placebo-controlled, randomized trial of Mingjing granule as an add-on to intravitreous ranibizumab for nAMD. One hundred eighty nAMD patients from six hospitals in China will be enrolled according to the inclusion and exclusion criteria and randomly allocated into two groups, 90 in each. All participants will receive a 24-week treatment and then be followed up for another 24 weeks. The primary outcome is the mean change of best-corrected visual acuity at week 24 and 48 as compared to the baseline. The secondary outcomes include mean change in central retinal thickness, area of retinal hemorrhage and exudation, and TCM syndrome score, mean number of intravitreal ranibizumab injection, and total cost of the treatment. Indexes of safety include blood regular test, urine regular test, liver function test, renal function test, and electrocardiogram from baseline to weeks 24 and 48. Qualitative control and some standard operating processes will be formed throughout the trial. Any ocular or systemic adverse events will be treated suitably, and related data will be recorded accurately and completely in the case report form.

**Discussion:**

Based on previous empirical and animal laboratory studies, this study will address the question of whether Mingjing granule could contribute to improving efficacy, safety, and efficiency with need for fewer intravitreal injections of anti-VEGF, improving compliance and visual outcomes in the management of persons with nAMD.

**Trial registration:**

Chinese Clinical Trial Registry (http://www.chictr.org.cn), ChiCTR2000035990. Registered on 21 August 2020.

**Supplementary Information:**

The online version contains supplementary material available at 10.1186/s13063-021-05025-x.

## Background

Age-related macular degeneration (AMD) is a progressive, degenerative disorder that in the late stage causes a large scotoma in the central visual field that leads severe visual impairment and ultimately blindness. AMD accounts for 8.7% of all blindness worldwide and is the most common cause of blindness in developed countries, particularly in people older than 60 years. Its prevalence is likely to increase as a consequence of exponential population aging, and the projected number of people will increase to 288 million in 2040 [1]. In large Asian ethnic populations as in China, the incidence of AMD is also increasing. The Jiangning Eye Study, a population study in a local community of Shanghai showed that the prevalence of early AMD in persons aged 50 years or older was 9.5% and that of late AMD was 1.0% [2].

According to the new international consensus, AMD is defined into no obvious age-related changes, normal age-related changes, early AMD, intermediate AMD, and late AMD [3]. Late-stage AMD is further classified into two clinical forms: geographic atrophy and neovascular AMD (nAMD). nAMD is responsible for the most rapidly progressive form of AMD leading to central visual loss and will have an increasingly significant detrimental impact on quality of life among the elderly population [4, 5]. nAMD is characterized by the formation of choroidal neovascularization (CNV), the ingrowth of new blood vessels from the choriocapillaris through Bruch’s membrane into the subpigment epithelial or subretinal space [6]. Anti-VEGF agents are currently recommended as the first-line therapy for the treatment of nAMD when there is evidence of subfoveal neovascular lesions based on the notion of VEGF being a main driver of angiogenesis [7, 8]. Large multicenter randomized clinical trials have demonstrated regression of these lesions and subsequent improvement of central visual acuity in nAMD patients in a relative short term [9–12]. Although at present, the gold standard treatment for nAMD is anti-VEGF, this treatment requires repetitive intravitreal injections that carry the inherit risks of severe local adverse reactions and long term complications. Ocular inflammation, increased intraocular pressure, uveitis or less frequently endophthalmitis, retinal detachment, and retinal or vitreous hemorrhage are all serious ocular adverse events to anti-VEGF treatment [13, 14]. Others reported a lack (down to complete lack) of response with standard treatment patterns and even a decrease in treatment efficacy after repeated intravitreal injections [15].

From a public health perspective, the high cost of the anti-VEGF treatments is in conflict with the increasingly financially stressed health systems, setting reimbursement by payers as the major limiting factor. In middle- and low-income economies that do not offer employer public insurance schemes or subsidies, these treatments are often not accessible, and if they are, they must be paid out of pocket and thus contribute to increasing financial distress to patients and their families. A study from Singapore has estimated the economic burden of wet AMD in 2030 to be between 203.1 and 162.9 million US$ based a growth of 42% in the number of wet AMD cases by 2030 [16]. Furthermore, the burden associated with frequent clinic visit or individualized re-treatments by intravitreal injections over a long period of time for patients, caregivers, and physicians is significant and may lead to suboptimal outcomes because of adherence problems and underdosing [12, 17]. Large real-world studies demonstrated that nAMD patients generally lose vision within 2 to 5 years of diagnosis, despite anti-VEGF therapy [18]. Therefore, from an economic, patient management and public health perspective, there is an important unmet need to address potential alternative and complementary treatments to the current use of anti-VEGF in the management of nAMD [19].

In China, traditional Chinese medicine has been used to treat eye diseases for thousands of years. Although there was not a specific terminology for AMD in TCM classic books of ophthalmology, there were records of such kind of diseases described as metamorphopsia. Some candidate TCM formulas for AMD have been summarized through accumulated clinical experiences. Mingjing granule, a Chinese herbal compound composed of *Astmgali Radix (huangqi), Salviae Miltiorrhizae (danshen), Lycii Fructus (gouqizi), Ecliptae Herba (mohanlian), Cattail Pollen (puhuang), Cirsii Japonici Herba Carbonisata (daji)*, is developed from an empirical formula of a famous veteran ophthalmologist of TCM. Our previous in vitro studies demonstrated that Mingjing granule could protect human retinal pigment epithelium (hRPE) cell lines from apoptosis induced by blue light, and it could also enhance the phagocytosis of RPE cells at proper concentration [20, 21]. The animal studies showed that Mingjing granule had a certain inhibitory effect on CNV proliferation and could prevent CNV leakage. The mechanism may be related with down-regulating the level of VEGF, upregulating the level of pigment epithelium-derived factor (PEDF) and decreasing M2-like macrophages in the retina of Brown Norway (BN) rat model [22–25]. In addition, some studies have reported that bone marrow-derived cells (BMCs) not only participated in the composition of CNV cells, but also secreted some cytokines to affect the occurrence and development of CNV [26–30]. In our previous study, it was shown that Mingjing granule could inhibit the contribution of BMCs in the formation of CNV [31, 32]. Furthermore, several clinical studies have reported Mingjing granule could stabilize the vision loss or improve visual acuity of nAMD, decrease the subretinal fluid, and reduce the area of CNV leakage with no significant adverse events or laboratory abnormalities [33–37]. However, to date, no high quality clinical trials have been undertaken for Mingjing granule. In this study, we will examine the efficacy and safety of the Mingjing granule in the treatment of nAMD through a randomized, double-blind, placebo-controlled trial.

## Methods

### Trial design

**Fig. 1:**
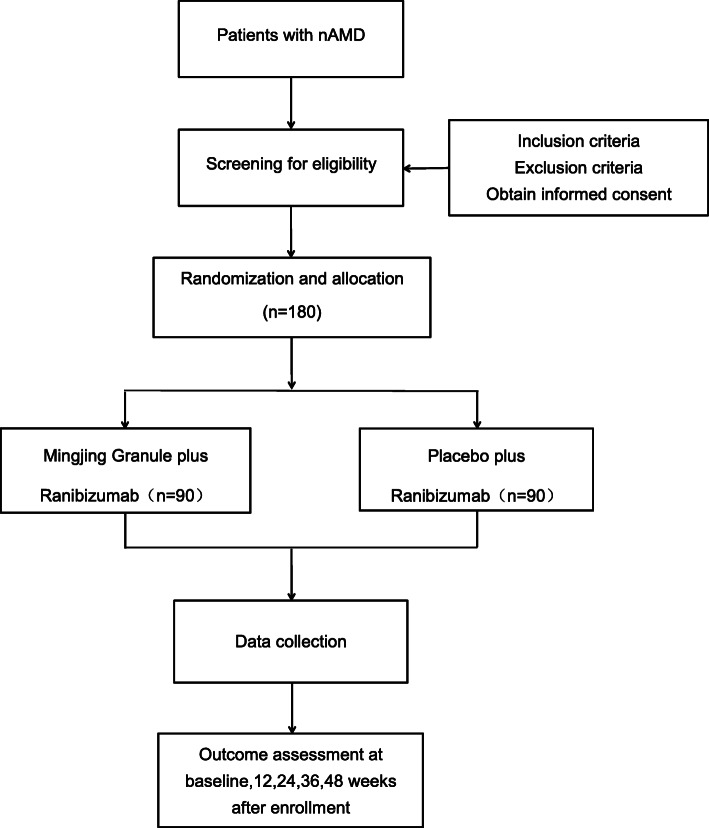
Trial flow chart. nAMD, neovascular age-related macular degeneration

**Fig. 2:**
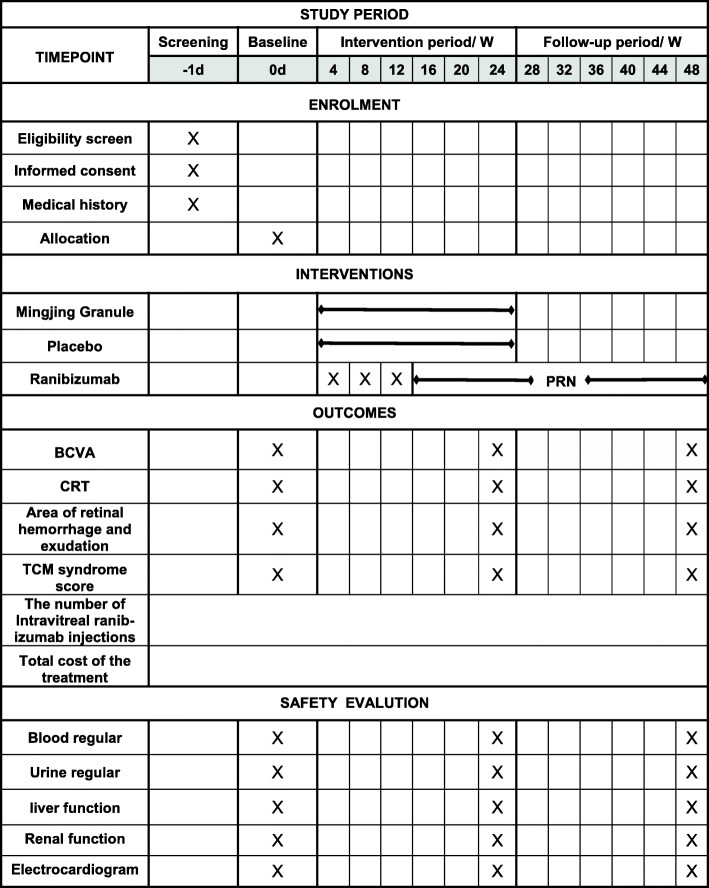
Study schedule. BCVA, best-corrected visual acuity; CRT, central retinal thickness; PRN, pro re nata. Ranibizumab will be administered as needed after three consecutive monthly injections (3 + PRN)

This is a randomized, double-blind, placebo-controlled trial. Participants will be randomly allocated into two groups, the test group and the control group. Patients in the test group will receive Mingjing granule plus ranibizumab, while those in the control group will receive placebo with ranibizumab. All of them will be followed up every 4 weeks. Mingjing granule and placebo will be dispatched every 4 weeks until week 24; ranibizumab will be administered as needed after three consecutive monthly injections until week 48 (Figs. 1 and 2). The protocol is designed following the guidelines of the Consolidated Standards of Reporting Trials and Standard Protocol Items: Recommendations for Interventional Trials (SPIRIT) (see Additional file 1 SPIRIT 2013 checklist). The trial has been registered with the Chinese Clinical Trial Registry (ChiCTR2000035990, registered on 21 August 2020).

### Trial setting

We will recruit 180 persons with diagnosed nAMD from six hospitals in China:

1. Foshan Hospital of Traditional Chinese Medicine,
2. Shenzhen Traditional Chinese Medicine Hospital,
3. Henan Province Hospital of Traditional Chinese Medicine/The Second Affiliated Hospital of Henan University of Chinese Medicine,
4. The Second Affiliated Hospital of Liaoning University of Traditional Chinese Medicine,
5. The Affiliated Hospital of Shandong University of Traditional Chinese Medicine/Shandong Province Hospital of Traditional Chinese Medicine,
6. The first Teaching Hospital of Tianjin University of Traditional Chinese Medicine.

### Recruitment

For the recruitment of possible participants for the study, we will advertise using an information poster placed on notice boards in every participating center and resident communities. The information poster will include a brief description of the subjects that are eligible and details of activities and necessary contributions as participants in the study. For those people who are ineligible or decline to participate, we will record the basic demographic information and reasons for non-participation. The investigator will introduce the protocol as well as the benefits and risks to the enrolled participants and an informed consent form is mandatory before the study.

### Randomization and allocation concealment

Random sequence table will be generated by the Strategic Applications Software (SAS, version 9.4, SAS Institute, Inc., Cary, USA) and performed by the Institute of Clinical Pharmacology, China Academy of Chinese Medical Sciences. Participants will be allocated randomly into one of the two groups with a ratio of 1:1. The identification code and random number is unique for each participant.

### Blinding

All participants, clinical physicians, nurses, data managers, and statisticians and other staff will be blinded to the treatment allocations until the trial is completed. The placebo looks and tastes indistinguishable from Mingjing granule. The randomization list and blinding codes will be kept strictly confidential. Emergency unblinding process should only be done when the knowledge of intervention allocation is essential to guide the clinical management. The blinding will be broken after obtaining the consent of principle investigator.

#### Inclusion criteria

1. Exudative changes due to active CNV lesions secondary to AMD in one eye or both eyes.
2. Aged 50 to 80 years.
3. The best-corrected visual acuity (BCVA) between 73 and 34 assessed by early treatment diabetic retinopathy study (ETDRS) charts (approximately 20/40-20/200 Snellen equivalent).
4. Agree to participate in the trial and provide written informed consent.

#### Exclusion criteria

1. Prior treatment of the study eye with intraocular anti-VEGF agents, verteporfin photodynamic therapy, other laser, corticosteroids, surgery (except cataract surgery at more than 30 days prior to screening) or systemic use of anti-VEGF products within 3 months prior to study entry.
2. Ocular condition in the study eye that might impact vision and confound study outcomes.
3. Active severe infection/intraocular inflammation in the study eye.
4. Unclear dioptric media in the study eye that affecting fundus photography and optical coherence tomography (OCT) examination.
5. Those with severe cardiovascular diseases, cerebrovascular diseases, impaired hepatic and renal function (aspartate transaminase/AST and/or alanine transaminase/ALT 2-fold the upper limitation of normal or above, the same for creatinine), hematopoietic diseases, or severe life-threatening primary diseases, mental illness (such as depression disorder, anxiety disorder).
6. History of allergy to the study/treatment-related agents.
7. Previous (within 3 months) or concomitant participation in another clinical study with investigational medicinal products.
8. Patients who the investigators deem to be ineligible for the study.

#### Withdrawal criteria

1. In cases of worsening disease or concomitant complications with a necessity of safety evaluation and curative treatment.
2. With the forbidden therapy and medication history during the trial, which will lead to bias for efficacy and safety evaluation.

### Interventions

#### Test group (Mingjing granule group)

Participants in the test group will be instructed to dissolve Mingjing granules (5.95 g/bag) in 200 ml hot water and take the solution orally twice a day for 24 weeks. Mingjing granule will be manufactured, packaged, and labeled by pharmaceutical factory (Beijing Tcmages Pharmaceutical Co. Ltd.). The main ingredients include *Astmgali Radix (huangqi), Salviae Miltiorrhizae (danshen), Lycii Fructus (gouqizi), Ecliptae Herba (mohanlian), Cattail Pollen (puhuang), and Cirsii Japonici Herba Carbonisata (daji)*.

#### Control group (placebo group)

For the control group, placebo contains 95% bitterant, edible lactose, starch, and pigment (such as lemon yellow, caramel pigment, and sunset yellow) and also includes 5% Mingjing granule to achieve the similar appearance, color, smell, taste, and weight as Mingjing granule.

#### Concomitant medications

Participants in both groups will receive 3 consecutive monthly intravitreal injections of ranibizumab (allowed to be injected at a dose of 0.5 mg per time). If necessary, they will be reinjected another during the followed up period. Decision for the reinjection will be guided on the criteria formulated by prospective OCT imaging of patients with neovascular AMD treated with intraocular ranibizumab (PrONTO) [38]: (1) a loss of 5 letters or more, (2) an increase in central retinal thickness (CRT) at least 100 μm, (3) new retinal hemorrhage appears, (4) new typical CNV, (5) fluid still exists after injection last month, and (6) any qualitative increase in the amount of fluid.

Participants will be requested not to receive any other treatments for nAMD, such as complementary treatments (e.g., herbal medicine, and acupuncture) or other medications, throughout this study. Participants will be allowed to take western medicine to treat the systematic diseases, such as hypertension or heart disease. The use of all drugs, if any, will be documented in detail in the case report form (CRF).

## Outcomes

### Primary outcome

#### The mean change of BCVA (from baseline to weeks 24 and 48)

We will assess the participants’ best-corrected distance visual acuity by ETDRS charts before and after the treatment at weeks 24 and 48. When necessary, the participants will be examined more than once.

### Secondary outcomes

#### The mean change of CRT (from baseline to weeks 24 and 48)

The participants’ eyes will be fully dilated; the examiner will take the macular fovea as the center and perform a multi-directional, 6 mm-long, radial linear scan of the most prominent macular lesions and take linear scans in the same direction after the treatment. CRT is defined as the distance between internal limiting membrane and external limiting membrane, excluding the fluid under retinal pigment epithelium (RPE). When necessary, the participants will be examined more than once.

#### Retinal hemorrhage and exudation (from baseline to weeks 24 and 48)

The participants’ eyes will be fully dilated; the examiner will take the fundus photography and calculate the area of retinal hemorrhage and exudation through Image Pro Plus software. Participants’ optic disc area will be used as a record before and after the treatment at weeks 24 and 48. When necessary, the participants will be examined more than once.

#### TCM syndrome score (from baseline to weeks 24 and 48)

Evaluation of TCM syndrome refers to clinical research guidance of new investigational drug in TCM. TCM syndrome score from baseline to weeks 24 and 48 will be calculated. The scores and details of TCM syndrome are given in Table 1.

**Table 1:** TCM syndrome score

Symptoms	Score of TCM symptom			
	None 0	Mild 1	Moderate 2	Severe 3
Dizziness and tinnitus	None	Occasionally	Sometimes, relieving after some rest	Often, still existing after some rest
Vexing heat in chest, palms, and soles	None	Vexing heat in palms and soles at night	Vexing heat in palms and soles day and night	Vexing heat in chest, palms and soles, and unwilling to cover the quilt
Soreness and weakness of waist and knees	None	Occasionally, not affecting daily life	Sometimes, weakness of legs and affecting daily life	Often, difficult to walk and affecting daily life
Dry mouth and throat	None	Slightly	Seriously	Seriously, and need some water
**Score:**				
**Tongue and pulse:**				

#### The mean number of intravitreal ranibizumab injections

We will calculate the mean number of intravitreal ranibizumab injections at week 48.

#### Total cost of the treatment

We will calculate the total cost of the participants’ treatment at week 48.

### Safety evaluation

We will monitor blood regular test, urine regular test, liver function test (AST, ALT, alkaline phosphatase/ALP, gamma-glutamyl transpeptidase/γ-GT, total bilirubin/TBIL), renal function test (creatinine/Cr, urea), and electrocardiogram (ECG) to certify whether Mingjing granule could be safe. Patients will be asked to report to the researchers any abnormal reactions occurring in the trial. All details of related and unexpected adverse events, such as time of occurrence, degree and duration, suspected causes, and the effective measures and outcomes will be recorded on CRF. Any ocular or systemic adverse events will be treated suitably and recorded accurately and completely as well.

### Ethics and dissemination

#### Patient consent and dissemination policy

This trial will be complied with the Declaration of Helsinki and Ethical Guidelines for Clinical Research. We will rigorously follow the latest Consolidated Standards of Reporting Trials (CONSORT 2017). The protocol has been permitted by the Research Ethical Committee of Eye Hospital, China Academy of Chinese Medical Sciences, Foshan Hospital of Traditional Chinese Medicine, Shenzhen Traditional Chinese Medicine Hospital, Henan Province Hospital of Traditional Chinese Medicine/The Second Affiliated Hospital of Henan University of Chinese Medicine, The Second Affiliated Hospital of Liaoning University of Traditional Chinese Medicine, The Affiliated Hospital of Shandong University of Traditional Chinese Medicine/Shandong Province Hospital of Traditional Chinese Medicine, and The First Teaching Hospital of Tianjin University of Traditional Chinese Medicine. The results of this study will be disseminated to the public through academic conferences and peer-reviewed journals.

#### Access to data and confidentiality

Privacy of participants will be highly respected in the conduct of the study. The personal information about the potential and enrolled participants will be collected to be used only in this trial. All data will be kept strictly confidential at the study site. Only research team members will have access to the research data. All local databases will be secured with password-protected access systems. Any data required to support the protocol can be supplied on request. Forms, lists, appointment books, and any other listings that link participant ID numbers to other identifying information will be stored securely with limited access. The information of participants will not be released and shared without the written permission of the participant.

#### Sample size

We design a superiority trial with a ratio of 1:1. The primary outcome is the mean change of BCVA at week 48 as compared to the baseline. After reviewing some literature, the mean change of BCVA in the test group is expected to increase by 10.1 letters measured by ETDRS chart, which in the control group is expected to increase by 8.2 letters [39, 40]. *n*_*c*_ is the participants’ number in each group. σ (standard deviation of the two groups) = 4. *Z*_1−*α*_ = 1.96, when *α* = 0.025; *Z*_1−β_ = 0.84, when *β* = 0.2. Δ = 0, *K* = 1, μ_T_ = 10.1, μ_C_ = 8.2. We calculate it by the PASS software; there are 71 cases in each group. Considering a 20% drop-out rate, there are 89 cases in each group and a total of 178 cases in both groups. Thus, we plan to recruit 180 participants in total (30 cases in every center, 15 cases in each group). The sample size calculation formula is as follows [41]:

$$ {n}_c=\frac{{\left({Z}_{1-\upalpha}+{Z}_{1-\upbeta}\right)}^2{\sigma}^2\left(1+1/K\right)}{{\left({\mu}_T-{\mu}_C-\Delta \right)}^2} $$

#### Statistical methods

All the efficacy and safety analyses will be conducted within the full analysis set (FAS) according to the intention-to-treat principle, with all randomly assigned participants included. Last-observation-carried-forward method will be applied in the missing values. We will conduct the per-protocol set (PPS) analysis to compare the results from FAS and PPS, and safety set (SS) will be used for the safety evaluation. Data from the six centers will be combined for statistical analysis of the primary and secondary outcomes as well as adverse events. Demographic, eye examination, and laboratory characteristics will be calculated at baseline, intervention, and follow-up period. The mean ± standard deviation will be chosen for continuous variables and the comparison between the two groups will be analyzed by *t* tests with normal distribution. Non-parametric Mann-Whitney-Wilcoxon test will be used for the comparison of data with non-normal distribution. Categorical variables will be compared using *χ*^2^ statistics, while the Fisher exact test will be used when the theoretical frequency is less than 5 in more than 25% of the cells. In order to control the center and baseline effects, covariance analysis will be applied for the intergroup comparison with continuous variables and Cochran-Mantel-Haenszel test for categorical variables. All statistical tests are unilateral tests; *P* < 0.05 means statistically significant. All statistical analyses will be performed by using SPSS V.19.0.

### Quality supervision and management

#### Request for researchers

Researchers or physicians will receive pre-clinical systematic training and get a full understanding of the protocol details, and they should possess the qualifications and ability to carry the study on and not be changed constantly.

#### Quality control of laboratory

Every center should offer researchers suitable medical equipment and emergency facilities. Laboratory, Medical Examination Center and other related departments will be established quality control and uniform standard operating processes. A certain item will be in charged by a special person.

#### Measures for study/treatment agents and compliance of participants

Mingjing granule and placebo will be manufactured by the same pharmaceutical factory and distributed by a trained staff in each center. Compliance of participants will be inspected by the method of drugs notation, and they will be asked to bring drugs last time in order to calculate the number. The formula is as follows:

$$ \mathrm{Compliance}=\left[\left(\mathrm{dispensing}-\mathrm{remaining}\right)/\mathrm{dispensing}\right]\times 100\% $$

#### Monitoring and inspection

A trial coordinating group will be established, which is responsible for the implementation of the trial, and they will visit each center regularly to check the progress of the trial, examine CRF, verify the storage of investigational agents, and record of data.

## Discussion

The medicinal herbal compound (fufang) is the most commonly used treatment approach of TCM in China. To the best of our knowledge, there are few high quality clinical studies that investigate the efficacy and safety of herbal compound in the treatment of AMD. Compared with previous studies [33–37, 39], this trial has more clinical research centers, clearer diagnostic criteria, a more rigorous methodology, and longer follow-up period.

Though the exact pathogenesis remains unclear, it is generally accepted that AMD is multifactorial disease related to environmental factors, genetic background, and parainflammation. Recent studies have found that autophagy dysfunction in RPE cells, cellular senescence, and abnormal immune-inflammatory responses play key roles in the pathogenesis of AMD. Neovascularization is an intermediate pathological phenomenon of nAMD; intravitreal injection of anti-VEGF agents is not an etiological treatment. It is easy to relapse after drug withdrawal [42]. Due to the multifactorial and complex nature of the disease, it may delay the process of AMD more effectively and obtain better clinical efficacy if multi-target effect could be achieved simultaneously. Unlike the single ingredient of western medicine, the composition of compound formula is very complex. Generally, a TCM compound formula is composed of several herbs; each herb normally has many ingredients, and multiple ingredients simultaneously act on multi-targets; a formula is a complex biologic active network [43].

Mingjing granule consists of Radix Astragali (huangqi), *Salvia miltiorrhiza* root (danshen), *Lycium barbarum* (gouqizi), *Eclipta alba* (mohanlian), Pollen Typhae (puhuang), and *Cirsium japonicum* (daji). Each herb in the formula has its bioactivities. For example, Radix Astragali is one of the most widely used traditional Chinese herbal medicines. It is used as immune stimulant, tonic, antioxidant, hepatoprotectant, diuretic, antidiabetic, anticancer, and expectorant [44]. *Salvia miltiorrhiza* root has been used for clinical treatment of various microcirculatory disturbance-related diseases for a long time. It contains many active water-soluble compounds, including protocatechuic aldehyde, 3,4-dihydroxyphenyl lactic acid, and salvianolic acid B. These compounds, as well as water-soluble fraction of *Salvia miltiorrhiza* root extract (SMRE), have an ability to scavenge peroxides and are able to inhibit the expression of adhesion molecules in vascular endothelium and leukocytes. Moreover, lipophilic compounds of SMRE also prevent the development of vascular damage [45]. *Lycium barbarum* (LB) is a renowned functional food and medicinal plant in Southeast Asia. Research data suggested that LB may exert neuroprotective effects and may aid in preventing neurodegenerative disease [46]. According to TCM theory raised from thousands of years of clinical practice, compatibility of medicinal herbs can strengthen the therapeutic efficacy and attenuate the toxicity of major component through drug-drug interaction. In this study, we will examine the efficacy and safety of Mingjing granule for nAMD patients. Our previous studies have shown that Mingjing granule could inhibit laser-induced CNV formation and development in BN rats through multi-targeting effects. Results from the pilot clinical study also showed that Mingjing granule could stabilize the patients’ visual acuity and reduce the area of retinal hemorrhage and exudation. And, no adverse effects have been observed through its clinical use, and toxicology studies in mice and rats support the safety of the proposed dose. These data support Mingjing granule as an alternative and complimentary therapy for CNV-associated disorders.

In summary, this protocol describes a randomized controlled trial conducted in 6 centers to evaluate the efficacy and safety of Mingjing granule in the treatment of AMD. If results of this trial demonstrates that Mingjing granule combined with ranibizumab has better efficacy, less injection times, and lower cost and good safety, it will provide useful clinically information for AMD and offer patients and ophthalmologists a new treatment option.

### Trial status

The updated version of trial protocol (version 2.0, 4 August 2020) has been reviewed and approved on 12 August 2020(YKEC-KT-2020-009). Recruitment will start in October 2020 and is expected to finish in March 2021.

## Supplementary Information

**Additional file 1.** SPIRIT 2013 Checklist: Recommended items to address in a clinical trial protocol and related documents.

**Additional file 2.** Statement on recognition of ethical approval letter.

## Data Availability

The datasets analyzed during the current study are available from the corresponding author on reasonable request.

## Abbreviations

nAMD: Neovascular age-related macular degeneration
anti-VEGF: Anti-vascular endothelial growth factor
TCM: Traditional Chinese medicine
AMD: Age-related macular degeneration
CNV: Choroidal neovascularization
hRPE: Human retinal pigment epithelium
PEDF: Pigment epithelium-derived factor
BN: Brown Norway
BMCs: Bone marrow-derived cells
SPIRIT: Standard Protocol Items: Recommendations for Interventional Trials
BCVA: Best-corrected visual acuity
ETDRS: Early treatment diabetic retinopathy study
OCT: Optical coherence tomography
AST: Aspartate transaminase
ALT: Alanine transaminase
PrONTO: Prospective OCT imaging of patients with neovascular AMD treated with intraocular ranibizumab
CRT: Central retinal thickness
CRF: Case report form
RPE: Retinal pigment epithelium
ALP: Alkaline phosphatase
γ-GT: Gamma-glutamyl transpeptidase
TBIL: Total bilirubin
Cr: Creatinine
ECG: Electrocardiogram
FAS: Full analysis set
PPS: Per-protocol set
SS: Safety set
SMRE: *Salvia miltiorrhiza* root extract
LB: *Lycium barbarum*
