# Supplementary material for: Mingjing granule, a traditional Chinese medicine in the treatment of neovascular age-related macular degeneration: study protocol for a randomized controlled trial
Source: Trials. 2021 Jan 19;22:69. doi: 10.1186/s13063-021-05025-x (PMC7814434; doi:10.1186/s13063-021-05025-x)
Supplement: Supplementary file 2 — Additional file 2. Statement on recognition of ethical approval letter. [file 13063_2021_5025_MOESM2_ESM.pdf]

关于认可伦理审批件的声明

本单位参与由国家中医药管理局资助课题：2019 中医药循证能力建设项目（编号：2019XZZX-YK002）。该课题已通过技术指导单位（中国中医科学院眼科医院）伦理委员会审批（编号：YKEC-KT-2020-009）和本单位伦理审批（批件号：2020-086）。我们将获得所有参与者的知情同意。

参与单位：佛山市中医院

Statement on recognition of ethical approval letter

This unit participated in a project funded by National Administration of Traditional Chinese Medicine: 2019 Project of Building Evidence Based Practice Capacity for TCM (No.2019XZZX-YK002). This subject has been approved by the ethics committee of the technical guidance unit (Eye Hospital, China Academy of Chinese Medical Sciences) (No. YKEC-KT-2020-009), and the ethics committee of the unit (Foshan Hospital of Traditional Chinese Medicine) (No. 2020-086). We will obtain the informed consent from all study participants.

Participating centre: Foshan Hospital of Traditional Chinese Medicine

关于认可伦理审批件的声明

本单位参与由国家中医药管理局资助课题：2019 中医药循证能力建设项目（编号：2019XZZX-YK004）。该课题已通过技术指导单位（中国中医科学院眼科医院）伦理委员会审批（编号：YKEC-KT-2020-009）。本单位承诺该伦理审批件适用本单位开展此研究，并将获得所有参与者的知情同意。

参与单位：深圳市中医院

深圳市中医院 医学伦理委员会

Statement on recognition of ethical approval letter

This unit participated in a project funded by National Administration of Traditional Chinese Medicine: 2019 Project of Building Evidence Based Practice Capacity for TCM (No.2019XZZX-YK004). This subject has been approved by the ethics committee of the technical guidance unit (Eye Hospital, China Academy of Chinese Medical Sciences) (No. YKEC-KT-2020-009). We promises that the ethical approval document is applicable for the unit to carry out this research and will obtain the informed consent from all study participants.

Participating centre: Shenzhen Traditional Chinese Medicine Hospital

关于认可伦理审批件的声明

本单位参与由国家中医药管理局资助课题：2019 中医药循证能力建设项目（编号：2019XZZX-YK005）。该课题已通过技术指导单位（中国中医科学院眼科医院）伦理委员会审批（编号：YKEC-KT-2020-009）。本单位承诺该伦理审批件适用本单位开展此研究，并将获得所有参与者的知[illegible]

参与单位：河南省中医院（河南中医药大学第二附属医院）

Statement on recognition of ethical approval letter

This unit participated in a project funded by National Administration of Traditional Chinese Medicine: 2019 Project of Building Evidence Based Practice Capacity for TCM (No.2019XZZX-YK005). This subject has been approved by the ethics committee of the technical guidance unit (Eye Hospital, China Academy of Chinese Medical Sciences) (No. YKEC-KT-2020-009). We promises that the ethical approval document is applicable for the unit to carry out this research and will obtain the informed consent from all study participants.

Participating centre: Henan Province Hospital of Traditional Chinese Medicine (The Second Affiliated Hospital of Henan University of Chinese Medicine)

关于认可伦理审批件的声明

本单位参与由国家中医药管理局资助课题：2019 中医药循证能力建设项目（编号：2019XZZX-YK008）。该课题已通过技术指导单位（中国中医科学院眼科医院）伦理委员会审批（编号：YKEC-KT-2020-009）。本单位承诺该伦理审批件适用本单位开展此研究，并将获得所有参与者的知情同意。

参与单位：辽宁中医药大学附属第二医院

Statement on recognition of ethical approval letter

This unit participated in a project funded by National Administration of Traditional Chinese Medicine: 2019 Project of Building Evidence Based Practice Capacity for TCM (No.2019XZZX-YK008). This subject has been approved by the ethics committee of the technical guidance unit (Eye Hospital, China Academy of Chinese Medical Sciences) (No. YKEC-KT-2020-009). We promises that the ethical approval document is applicable for the unit to carry out this research and will obtain the informed consent from all study participants.

Participating centre:The Second Affiliated Hospital of Liaoning University of Traditional Chinese Medicine

关于认可伦理审批件的声明

本单位参与由国家中医药管理局资助课题：2019 中医药循证能力建设项目（编号：2019XZZX-YK009）。该课题已通过技术指导单位（中国中医科学院眼科医院）伦理委员会审批（编号：YKEC-KT-2020-009）。本单位承诺该伦理审批件适用本单位开展此研究，并将获得所有参与者的知情同意。

参与单位：山东中医药大学附属医院/山东省中医院

Statement on recognition of ethical approval letter

This unit participated in a project funded by National Administration of Traditional Chinese Medicine: 2019 Project of Building Evidence Based Practice Capacity for TCM (No.2019XZZX-YK009). This subject has been approved by the ethics committee of the technical guidance unit (Eye Hospital, China Academy of Chinese Medical Sciences) (No. YKEC-KT-2020-009). We promises that the ethical approval document is applicable for the unit to carry out this research and will obtain the informed consent from all study participants.

Participating centre:The Affiliated Hospital of Shandong University of Traditional Chinese Medicine/Shandong Province Hospital of Traditional Chinese Medicine

关于认可伦理审批件的声明

本单位参与由国家中医药管理局资助课题：2019 中医药循证能力建设项目（编号：2019XZZX-YK0012）。该课题已通过技术指导单位（中国中医科学院眼科医院）伦理委员会审批（编号：YKEC-KT-2020-009）。本单位承诺该伦理审批件适用本单位开展此研究，并将获得所有参与者的知情同意。

参与单位：天津中医药大学第一附属医院

Statement on recognition of ethical approval letter

This unit participated in a project funded by National Administration of Traditional Chinese Medicine: 2019 Project of Building Evidence Based Practice Capacity for TCM (No.2019XZZX-YK0012). This subject has been approved by the ethics committee of the technical guidance unit (Eye Hospital, China Academy of Chinese Medical Sciences) (No. YKEC-KT-2020-009). We promises that the ethical approval document is applicable for the unit to carry out this research and will obtain the informed consent from all study participants.

Participating centre:First Teaching Hospital of Tianjin University of Traditional Chinese Medicine

天津中医药大学第一附属医院眼科
